# Infant disorganized attachment: Clarifying levels of analysis

**DOI:** 10.1177/1359104516685602

**Published:** 2017-02-01

**Authors:** Robbie Duschinsky, Judith Solomon

**Affiliations:** Institute of Public Health, Cambridge University, UK

**Keywords:** Disorganization, attachment, classification, conflict behavior

## Abstract

Lack of clarity regarding the infant disorganized attachment classification has caused confusion in the clinical, forensic, and research contexts in which it is used. This article offers distinctions to clarify the concept with the goal of increasing understanding and identifying potential misapplications. In particular, attention is drawn to the fact that there are many indices used to code “disorganized attachment,” and that so far they have been validated as a set rather than individually; and it is noted that the construct validation of disorganization in naturalistic settings is partially finished. Clinicians and social workers should be cautious in their interpretations of such behavior.

## Introduction

Three classifications for infant behavior in the Strange Situation were introduced by Ainsworth, Blehar, Waters, and Wall (1978). These distinct patterns of behavior were understood to represent strategies for achieving the physical and attentional availability of the caregiver and to vary as a function of the caregiver’s responsiveness to the child’s signals of distress. An additional, insecure-disorganized/disoriented (D) classification for coding the Ainsworth Strange Situation was collaboratively introduced by one of the authors of the present article (Main & Solomon, 1990). The classification was based on the observation of sometimes brief, out-of-context, unexpected, or anomalous behaviors, which suggested disorientation, fear in relation to the caregiver, or a high level of conflict about approach to the caregiver. The D classification has been widely assumed to represent undifferentiated chaos; in Spangler and Schieche (1998), for instance, the authors write that “as disorganized infants, *by definition*, do not have any coherent strategies, behavioral regulation is restricted or even not possible at all” (p. 700). Likewise, other researchers describe disorganization as the “lack of any strategy” or the “lack of any way of coping with stress,” and theorize on this basis. To take an example from a recent and important article, “disorganization is defined as the collapse of attachment strategy under conditions of stress; under such conditions, disorganized individuals select a set of behaviors that are irrelevant to their need for downregulation of discomfort” (Wazana et al., 2015, p. 1157).

The chapters by Main and Solomon (1986, 1990) have served as a guidepost, prompting a good deal of significant developmental attachment research. Yet as is common in the history of science (Hacking, 2004), subsequent findings and usages point to the need for clarifications to avoid reification of the original construct. The need for such clarifications of the account presented in the Main and Solomon chapters is especially pressing in the context of calls in recent years from attachment researchers and clinicians for further consideration of what has been captured by the concept of “disorganized” attachment (see, for example, Beeney et al., 2017; Bernier & Meins, 2008; Hollidge & Hollidge, 2016; Lyons-Ruth et al., 2013; Padrón, Carlson, & Sroufe, 2014; Slade, 2014; Spieker & Crittenden, 2010). Tarren-Sweeney (2014) has argued that “further research is needed to understand the nature and clinical meaning of the disorganised attachment category” (p. 334). In the latest edition of the *Handbook of Attachment*, Lyons-Ruth and Jacobvitz (2016) urge that, for the field going forward, the behavior and processes of disorganized attachment will need to be considered carefully. For instance, Lyons-Ruth and Jacobvitz propose, recognising that this could be controversial, that it will be “important to narrow the current research criteria for disorganization to include only the forms to be considered disordered.”

In short, it is our view that some conceptual housekeeping is necessary. In attempting this, we have drawn inspiration from the classic work of Masterman (1970). Masterman identified 21 different uses of the term “paradigm” in the work of Thomas Kuhn and showed how this confusion led discussions of Kuhn’s work profoundly astray and blocked avenues for effective use of the concept. Masterman logically differentiates the uses of the term “paradigm” to specify the different phenomena, which the concept variously draws attention to—but then heaps together. Kuhn (1970) described Masterman’s work as helping to identify connections and unravel confusions, which would not “likely be apparent to anyone who has taken the notion of paradigm less seriously than Miss Masterman, for, as she quite properly emphasizes, I have used the term in a number of different ways” (p. 271). These distinctions would go on to be used by those seeking to do precise work with Kuhn’s characterization of science, including subsequently by Kuhn himself—and by John Bowlby (e.g. Bowlby, 1982, p. 668) in characterizing the kind of paradigm represented by attachment theory. We argue in this article that the term “disorganization” can refer to events and constructs at different levels of analysis, and that these are too often confused. In doing so, we hope to move discussions of “disorganization” to firmer conceptual ground, and support more critical and thoughtful usage of the classification by clinicians and researchers.

## The origin and significance of “attachment disorganization”

In Bowlby’s (1969) account of attachment, under typical rearing conditions human infants become motivated to approach their primary caregivers when alarmed, tired or ill, or following a separation. Bowlby presented the construct of an “attachment behavioral system” or “attachment system” to explain the operation and development of this motivational tendency. This system regulates attention, affect, and behavior, a process that includes recruiting and coordinating expectations based on past events, in order to achieve the physical and attentional availability of the caregiver. Once this goal is achieved, the attachment system becomes quiescent, permitting the infant to pursue other ends, such as exploration of the environment or affiliation with friendly people, though the infant will continue to monitor the environment for potential threats and for the whereabouts of their caregiver.

Bowlby’s account of the attachment system was used to conceptualize infant behavior in the Ainsworth Strange Situation (Ainsworth et al., 1978). In this procedure, a caregiver takes leave of and returns to their infant twice in a novel environment with interesting toys: first leaving the child with a stranger and then alone. Three patterns of infant behavior were initially identified by Ainsworth and colleagues. After the separation episodes, most infants seek proximity with the caregiver immediately, calm if they have been distressed, and return to play within the first 1 or 2 minutes of the reunion. This pattern corresponds to Bowlby’s model of the direct expression of the attachment behavioral system, and was found by Ainsworth, as well as later by researchers, to reflect an infant’s experience of a familiar caregiver who responds to the infant’s signals of distress with prompt and sensitive contact and soothing. Ainsworth termed the pattern “secure” (B). Infants classified as “avoidant” (A) initially direct their attention and orient away from the caregiver, regulating themselves through a focus on toys, though most will show a delayed or partially-suppressed inclination to approach. Ainsworth observed that at home, the caregivers of these infants were frequently seen to rebuff physical contact or to be insensitively intrusive. She theorized that infants displaying the avoidant attachment pattern could avoid the distress of rejection or uncomfortable interaction with their caregiver (Ainsworth et al., 1978; see also Main 1979 Isabella & Belsky, 1991). Infants classified ambivalent/resistant (C) utilize displays of anger and/or passive, helpless distress to maintain the attentiveness of a caregiver who, at home, tended to delay responding or whose availability might be inconsistent. Though relatively stable over time, researchers have found that an infant’s attachment pattern may alter in predictable ways if there are changes in the caregiving environment they experience (Sroufe, Egeland, Carlson, & Collins, 2005).

A fourth Strange Situation classification, “disorganized/disoriented,” was added by Main and Solomon (1986, 1990). Based on their observations of normative, high-risk, and maltreatment samples, Main and Solomon suggested that distinctive features of disorganization could be captured using seven broad “indices” of behavior based on morphology:

Sequential displays of contradictory behavior;
Simultaneous display of contradictory behavior;
Undirected, misdirected, or incomplete movements;
Stereotypies, mistimed movements, and anomalous postures;
Freezing or stilling;
Display of apprehension of the caregiver;
Overt signs of disorientation or disorganization.

Under each of these headings, Main and Solomon listed exemplars drawn directly from observation. To guide the coder toward a classification decision, some of these exemplars were placed in *italics* as, on their own, sufficient warrant for an overall D classification; others were not placed in italics, with the implication that several would need to accrue before the D classification should be considered. It was theorized that, to varying degrees, these different behaviors could be regarded as expressions of conflict at the level of the attachment system. As such, it was advised that a D classification should always be assigned, where possible, with a “best-fit” Ainsworth classification as the pattern for which “disorganization” represented a disruption. Main and Hesse (1990) suggested that one important and sufficient cause of conflict at the level of the attachment system, though not necessarily the only one, would be an infant’s experience of a caregiver who displays frightening or frightened behaviors toward them. They later added that other caregiver behaviours, such as dissociation, could have the same consequence of alarming the infant. Observations of parent–infant interaction have both confirmed and elaborated this hypothesis (Madigan et al., 2006). Relatedly, interviews with mothers of infants classified as disorganized/disoriented show that they experience themselves as helpless, that is, out of control or very emotionally dependent upon the child (Solomon & George 2011).

Since the D classification was introduced by Main and Solomon (1990), infants whose relationships have received this classification have been found to be substantially more common in at-risk samples (Cyr, Euser, Bakermans-Kranenburg, & van IJzendoorn, 2010), and the classification has proven predictive of later child mental health problems. Infants classified as disorganized/disoriented appear to have a markedly elevated risk of later externalizing disorders (*d* = .34, Fearon, Bakermans-Kranenburg, van IJzendoorn, Lapsley, & Roisman, 2010). Hygen, Guzey, Belsky, Berg-Nielsen, and Wichstrøm (2014) report that in their study, as in other samples, 85% of infants classified as disorganized/disoriented show either controlling-and-caregiving or controlling-and-punitive behavior to their attachment figure by age 6. Following Main and Cassidy (1988), who first observed these later attachment patterns, it is theorized that the controlling strategies are helping to render a parent’s caregiving more predictable even if, especially for the controlling-and-punitive child, there may be attendant negative consequences. On the basis of such predictive validity, strong claims have at times been made for the classification by later clinicians, social workers and researchers. Ballen, Bernier, Moss, Tarabulsy, and St-Laurent (2010) have observed that, “in the last decade, the field of developmental psychopathology has devoted increasing interest to what appears to be one of the most meaningful risk factors for later maladjustment: infant disorganized attachment” (p. 118).

## Revisiting “disorganization” in Main and Solomon

Wittgenstein (1980), among others, has observed that divergence between the use of a term in scientific psychology and in ordinary language is common; he noted that the nature of such divergence should, however, be identified if bedeviling confusion is to be avoided. Bowlby (1973) made much the same claim across his writings, warning that “it becomes easy for the unwary to assume that, because in common speech words are used without discrimination, whatever is referred to can be treated as though it were undifferentiated” (p. 118). In discussing the term “mourning,” for example, he notes that psychological usage is not quite the same as ordinary language but that the term has an encompassing quality which makes it “possible to link together a number of processes and conditions that evidence shows are interrelated” (Bowlby, 1979, p. 100). Bowlby urged that the fact that psychological processes are interrelated, however, should not lead researchers to fail to distinguish them conceptually and empirically.

Ainsworth (1972) had introduced “organization” as just such a technical term. In her usage, it served to describe the way the infant’s attention and behaviors were brought together to form a coherent pattern, which functioned smoothly as a whole to maintain the availability of the caregiver in the Strange Situation. The term “disorganization” therefore appealed to Main and Solomon as a way of thinking about discrepant infant behaviors observed in the Strange Situation. It was preferred to “disordered attachment,” an early alternative, as the term “disorganized” was considered less stigmatizing (see Duschinsky, 2015). Yet, looking back with the advantages of hindsight, it is clear that use of the term “disorganization” has caused confusion. A first and perhaps the overriding problem has been that, corresponding to Ainsworth’s technical use of the term “organized,” uses of the term “disorganized” differed from the dictionary definition—and that this was not identified to readers by Main and Solomon at the time, a fact that both authors now regret (Mary Main, personal communication). The dictionary, everyday meaning of the term “disorganization” suggests randomness and a lack of predictable responsiveness to contingencies: “to destroy the organization or systematic arrangement of; to break up the organic connection of; to throw into confusion or disorder” (Oxford English Dictionary, 2016). None of the senses given the word in Main and Solomon’s (1986, 1990) chapters were intended to imply this.

Yet, as well as departing from everyday uses of the term “disorganized,” in retrospect a second problem has been that the chapters introducing the classification used the term “disorganization” in different ways, which do not necessarily overlap. In particular, “disorganization” was used to describe both *observable behavior* and the invisible *psychological process* inferred from visible behavior. Thus, the same term was used as a characterization of two different levels of analysis:

1. *Contradiction or confusion in the morphology of observable behavior in the Strange Situation* e.g. “the most striking theme running through the list of recorded behaviors was that of disorganization or, very briefly, an observed contradiction in movement pattern” (Main & Solomon, 1990, p. 133), such as crying and approaching the caregiver but with head sharply averted;
2. *Disruption inferred to be occurring at the level of the infant’s attachment behavioral system, a psychological process* e.g. hand-to-mouth behavior on reunion as a “*direct* index of disorganization” (Main & Solomon, 1990, p. 139). This second usage somewhat resembles the dictionary definition, but is a description of the relative contradiction in attachment strategy (Main, 1990) or incoherence at the level of the behavioral system. It does not imply a stable state of chaos for the organism as a whole, which is suggested by the dictionary definition of “disorganization”: there was no presumption that the behaviours seen in the Strange Situation would be stable over time or shown by the infant similarly in their home environment.

The potentially misleading shift between the behavioural and the psychological uses of the term “disorganization” can be noted, for instance, in the description of “confused or confusing sequences of very rapid changes of affect in first few seconds of reunion with parent” as suggesting disorganization (Main & Solomon, 1990, p. 140). “Confused” sequences are observable in the infant. The term refers to behavior and movement that has the characteristic of being confused. By contrast, “confusing” sequences are those where the child’s goals are not clear to the observer. But both “confused” and “confusing” sequences are termed “disorganized,” because of the pivoting of this latter term in the Main and Solomon chapters. A vignette may help clarify the point:

Toby,15 months old, seen in the Strange Situation with his mother. Following a three-minute separation, his mother re-enters. Toby gets up and walks diagonally across her pathway towards the corner of the room, where he stands facing the join in the wall. The stranger heads out the door and closes it behind her. Toby makes his way to the stranger’s chair. As he gets there, he loses his balance. Sitting on the floor, he looks around the room, as if searching for the stranger. Toby’s scanning of the room alights on mother; his face darkens on seeing her. He then is quite still for ten seconds. There is no movement besides the rise and fall of his chest. Mother makes a comment about the toys to him. Toby gets himself up and makes an approach to his mother.

*Commentary*. On reunion with a caregiver following a brief separation, Bowlby’s theory suggests that the attachment system will dispose attachment behavior to retain the attentional and physical availability of the caregiver. Ainsworth’s most salient discovery was that this disposition may be blocked when an infant directs attention away from their attachment figure and toward the environment, a process which forms the avoidant (A) pattern. Toby does seem to have been stirred by his caregiver’s re-entry. However, his approach behavior, if approach it is, is not directed to his mother but sends him to the corner of the room. The behavior is serving to direct his attention away from the caregiver and toward the environment. It is therefore technically avoidant. But smoothly sequenced avoidance would send the child away from mother whereas a trajectory which runs diagonally across mother’s path suggests contradiction at the level of intention or plan. In the confusingly circular language used in the Main and Solomon chapters, Toby’s *disorganized behavior* is suggesting *disorganization of the attachment system*. In fact, however, the disruption of the visible sequencing of behaviour and the disruption of the invisible attachment system do not always correspond. This point can be illustrated by “misdirected behaviors” (Index III), such as trying to follow the stranger out of the room after being reunited with the caregiver. This isconsidered disorganized in the Main and Solomon chapters because it contradicts the expected behavioral output of the attachment system to seek proximity and the availability of the caregiver following a separation. Such misdirected behavior may actually be smoothly sequenced and show no “contradiction of movement pattern.” However, the behavior can be *inferred* to represent a disruption of the attachment system, and so suggest a contradiction or disruption “in intention or plan.”

In using the same term “disorganized” to refer to both behavior and psychological process, Main and Solomon had a specific aim, though it was not well articulated at the time. “Disorganization” was used as a conceptual tool for picking out “an observed contradiction in movement pattern, *corresponding* to an inferred contradiction in intention or plan” (1990, p. 133). The goal was to laminate (1) observable behavior and (2) psychological process at the level of imputed behavioral systems, with the latter being the ultimate focus of their attentions. This compression of meanings reflected their observation that no further discrete infant attachment “organizations,” in Ainsworth’s technical sense of the term, were apparent to them, and to justify what at the time was felt to be the “radical notion that the many, highly diverse indices of disorganization and disorientation can be placed under one heading” (1990, p. 151). The theoretical stakes of using the term “disorganized” to mean both behavior and psychological process was the claim that the diverse behaviors picked up by the Main and Solomon indices could well have different antecedents and sequalae, but what they had in common was that they suggested disruption or breakdown at the level of the attachment system.

As we have seen, the term “disorganization” was used in the Main and Solomon chapters to refer to a *contradiction in observed movement* pattern and to refer to some degree of breakdown at the level of the attachment system as a *psychological process*. Yet a third, distinct, usage of the term “disorganization” was in terms of *taxonomy* or classification. Where behavioral indices of disorganization are present, the Main and Solomon protocols indicate a coder should consider assigning the infant’s Strange Situation behavior to a D classification. To facilitate this task, Main and Solomon (1990) presented general guidelines and a 9-point scale without behavioral anchors for ranking how certain a coder is that they are seeing an interruption or breakdown of the attachment system, where five is sufficient for placement of the dyad into a D classification. On retrospective examination, the scale for coding disorganization as a taxonomic entity threads together some quite different characteristics of behavior. These are at most partially specified—a fact that has not only caused confusion about the classification but also contributed to difficulties for those training to code D and achieve reliability. However, the weightings can be gleaned from the commentary on the indices (Main & Solomon, 1990, p. 151), the brief descriptions that accompany the scale points, and detailed scrutiny of which behaviors are italicized. The weightings, retrospectively, appear to be as follows: (1) frequency of a behavior, (2) its pervasiveness or duration, (3) its abruptness or jerkiness in behavioral sequence, (4) the extent to which it occurs either close to reunion or in physical proximity with the caregiver, and (5) the extent to which it cannot be better explained as as a reaction to the immediate environment (e.g., stepping aside from the mother to avoid an obstacle on the floor). A sixth weighting, arising from Main and Hesse’s (1990) theory, is 6) the extent to which the infant’s responses to their caregiver suggest the experience of fear.

The term “disorganization” was therefore also used in a third sense, as a taxonomic label. So, as well as behaviour and psychological process, the same term was also used to refer to:

3. *Infants who scored 5 or more on the 1–9 scale* delineated in Main and Solomon (1990). The scale indicates the certainty with which the coder perceives that the behaviors they can observe are indicative of disruption of the attachment system.

These three different uses of the term “disorganized” are shown in Table 1.

**Table 1.** Uses of the term “disorganization” in Main and Solomon (1990).

Use of the term “disorganized”	D behavior	D system	D classification
Level of analysis	Observable behavior	Psychological process inferred from behavior pattern	Insecure-disorganized/disoriented classification
Refers to . . .	Behavior which appears sudden, out of context, disrupted, anomalous, contradicted, misdirected, frightened or disoriented.	Infant’s attachment system is disturbed or contradicted to a significant degree by a countervailing affect or intention.	A taxonomic label for a relationship in which substantial conflict at the level of the attachment system is inferred.
Illustrative quotation from Main and Solomon (1990)	“The most striking theme running through the list of recorded behaviors was that of disorganization” (p. 133)	“Support for the disorganizing effect of frightening behavior on the part of the parent was obtained in two recent studies of maltreated infants” (p. 124)	“The main purpose of the present paper is to formally present a set of indices . . . that will permit the identification of D” (p. 125)

To avoid confusion and to increase understanding of the meaning of disorganization and its clinical significance, distinctions are needed between these different levels of analysis. Specifically, research will be helped by the availability of a distinction between D as *an attachment classification* running orthogonal to the Ainsworth categories (distinguishable as *D-class*), D as the *behaviors listed in the Main and Solomon indices* (distinguishable as *D-behav*) and D as the *imputed disruption or disturbance of the attachment system* and its plan to gain the physical and attentional availability of the caregiver (distinguishable as *D-sys*).We do not anticipate that distinguishing D-class, D-behav, and D-sys will always be necessary when meaning is clear from the context, but anticipate they will be useful as a resource for thinking and for pin-pointing phenomena for discussion or inquiry. In particular, we hope that awareness of these different levels of analysis can reduce the widespread practice among researchers and clinicians of jumping from the word to conclusions, and the equally widespread practice of talking past one another about “disorganization” and causal processes, such as whether all infants showing D-behav are scared of their caregiver, are scared by the same maternal behaviors and to the same degree. It is also anticipated that these distinctions will be useful in sharpening recognition that the behaviors listed in the Main and Solomon indices may not always express disorganization at the level of the attachment system, as for instance in the case of autistic stereotypies (Granqvist et al., 2016).

To offer an example how these distinctions may be helpful in clarifying discussions, let us take the account in Hesse and Main (2000, 2006) of disorganization/disorientation as “the collapse in attentional and behavioral strategy.” This reference to a “collapse” of strategy has been widely misunderstood, with many presuming that disorganization/disorientation as a collapse in behavioral and attentional strategies always means a pervasive and chaotic display of observable behavior. This misunderstanding was unfortunately and inadvertently predisposed by the fact that a child physically collapsing to the floor was used as a privileged example in Main and Hesse’s texts of collapse at the level of strategy. In fact, however, the discussion of “collapse in attentional and behavioral strategy” was intended as a statement about the (invisible) attachment behavioral system (D-sys) and was not necessarily intended as a description of observable attachment behavior (D-behav). Depending on its extent, collapse in attentional and behavioral strategy could well, but may not, result in disorganized classification (D-class).

Similarly, the distinction between levels of analysis can help clarify the status of two of the Main and Solomon indices, which very frequently cause confusion to those learning to code. These two indices describe behavior which may, in fact, be coherently sequenced, understandable, and responsive to caregiver cues. It is not apparent, without distinguishing levels of analysis and a sharp awareness of the divergence from the dictionary definition, why such behaviors could be considered “disorganized”:

1. An observation of the infant hiding under a chair as the parent returns would be coded as a display of “apprehension of the caregiver” (Index VI). Even at the time, Main and Solomon (1990) expressed concerned awareness that “signs of apprehension may seem less disorganized or disoriented than many of the other behavior patterns” (p. 146)—the behavior may be smoothly enacted, without signs of conflict, and responsive to the caregiver’s past or present behavior. At another level, however, the behavior is disorganized because it indicates a powerful disruption of the attachment system (D-sys), which would be expected otherwise to induce an alarmed child to achieve proximity to the caregiver. Indeed as a strong indicator, this behavior might be sufficient to place the child into the “disorganized/disoriented” classification (D-class).
2. A similar jump across levels of analysis causes confusion in thinking about sequential contradiction of behavior between episodes (Index I). Crying desperately for the parent during separation followed at the moment of reunion by a blank expression and movement away (i.e. avoidance) does not necessarily appear as a conflict within the same behavioural sequence, but across sequences. Each individual sequence - whether crying, or avoiding - may be smoothly performed, responsive to environmental cues, and without other markers of tension or loss of regulatory control (Crittenden & Ainsworth 1989). Yet the behavior suggests some contradiction at the level of D-sys (Waters & Valenzuela 1999). To draw a comparison, a child following a familiar Ainsworth avoidant (A) attachment pattern *does* experience conflict between a desire to approach and to inhibit approach to the caregiver. However, the infant typically shows little or no distress in separation as long as he or she is not alone in the room, as attention is successfully directed away from the demands of the attachment system in a consistent way across episodes.

The difficulty these two examples present readers of Main and Solomon and those learning to code is not incidental but expresses something important about differences among D-behaviors. As Waters and Valenzuela (1999) argue, it is an empirical question whether Index I sequential contradictory behaviors and Index VI apprehension of the caregiver are two sets of behaviors occurring through the same psychological mechanisms, meaning the same thing or having the same sequelae. Whereas Wazana et al. (2015) assume, based on what the word disorganized means in ordinary language, that “disorganized individuals select a set of behaviors that are irrelevant to their need for downregulation of discomfort” (p. 1157), this may be an oversimplification. In distinguishing between levels of analysis, potential differences of degree and kind among D-behav come into view. Our suspicion is that they are related, but that they are not therefore the same. In an earlier study, Abrams, Rifkin and Hesse (2006) found that some forms of parental frightened/frightening behaviour predict disorganized attachment in the Strange Situation better than others, but can be theorized as part of a single construct. Similarly, we anticipate that Index I and Index VI both readily represent disruption of the attachment system (D-sys), but that much could be learnt by considering their particular antecedents, concomitants and sequalae. This raises a question that researchers are only beginning to address.

## Implications and applications

We have argued that partly as a product of Main and Solomon’s wording and partly the result of misunderstandings of their writings, there has been a conflation of the behavioral, systems, and taxonomic levels of analysis in using the term. This has been compounded by reification of Main and Hesse’s “fear without solution hypothesis” as suggesting that all infants classified as disorganized are afraid of their caregiver in the same way. The resulting desiccation of the concept of disorganization has supported accounts of disorganized/disoriented attachment behavior as an *undifferentiated set*, caused by disorganization/disorientation as a *unitary process* (see, for example, Cummings, 1990; Gergely, 2004). Such assumptions have not just led to linguistic ambiguities but misdirected clinicians and researchers.

To take clinical practice and research in turn: many examples of clinicians reading Main and Solomon’s chapters through the lens of the everyday connotations of “disorganization” and conflating levels of analysis could be given. One influential case of an interpretation along these lines is Brown and Ward (2013), a text by two British psychologists which appealed to the authority of work on disorganized attachment in providing the mandate for a radical (and controversial) shift in how proceedings to take children into care operate within the British family courts (see Holt & Kelly, 2016). Brown and Ward assert that attachment disorganization occurs when infants are:

fearful of approaching their caregivers because they cannot predict the response: sometimes they may be picked up and cuddled, but at other times they may be shouted at or smacked. As a result, these children are not able to “organize” their own behavior, and . . . behave unpredictably.

For Brown and Ward, then, “disorganization” means that unpredictability without logic in the parent breeds unpredictability without logic in the child, mediated by the infant’s fear of their caregiver. By conflating disorganization, unpredictability and harm, the authors leap to unwarrented conclusion. Brown and Ward imply that clinicians who see any disorganized attachment behaviors should therefore regard a child as at a great deal of risk, and court procedure must be changed to act fast and drastically. Similarly, Rees (2011) has urged pediatricians that “disorganized patterns arise if pervasive abuse leaves children ineffective both in self-sufficiency and in using relationships, lacking understanding of their own and others’ feelings. Safe independence is unlikely and criminality in adulthood common without recovery” (p. 187). In statements like this, clinicians and researchers appear to ride assumptions about the term “disorganization” to hasty conclusions. And it is a short step from the notion of disorganization as fearful chaos to the misapprehension of a D classification as a marker of pathology, in itself requiring social services intervention. However, this does not mean that the idea of disorganization lacks clinical significance, or that he Strange Situation cannot have relevance in clinical assessment. For instance, considering the possibility of contradictions between intentions of behavioral plans, and thinking differentially what might be causing this, is valuable for clinicians, as it can help orient us in interpreting behavior.

Similarly, confusion about the concept of “disorganization” among researchers has materially shaped the field’s methodology, such as over-reliance on a simple D/non-D classification, and throwing away data on disorganized behaviors where these do not reach the D-class threshold. It is notable that where researchers have found contradictory results, as for instance with the relationship between DRD4 repeat polymorphism and disorganization (e.g. Wazana et al., 2015) or the relationship between disorganization and later dissociative behaviors (e.g. Haltigan & Roisman, 2015), it does not appear to even be considered that the anomalous results could be a consequence of *different forms* of disorganization differentially predominating in the respective samples. Yet in the three decade since Main and Solomon (1986) we have not seen a single lab report or even mention of the distribution of D indices in their sample. Nor has any lab sought to examine the consistency of expressions of disorganized behavior shown by a child from one Strange Situation to a second. The behaviors have become invisible to the field, with attention paid exclusively to the classification and its correlates.

It should be acknowledged that there was something of a tendency in the work of Main and colleagues in the early 1990s to depict disorganization as undifferentiated chaos. Generally today researchers do not discuss this: they just assume it. There are, however, some who have explicitly claimed that disorganization represents undifferentiatedly meaningless behavior (e.g. Beebe & Lachmann, 2014; Daniel, 2015; DeOliveira et al., 2004). For these researchers, an important piece of evidence is Spangler and Grossmann (1993), who report that the infants classified as D in their sample had a distinct, elevated heart-rate pattern. However, Spangler and Grossmann (1999, p. 102) later acknowledged that the overwhelming majority of the association between D and heart rate was attributable to infants who showed Index VII behavior (“direct indices of disorganization”), and there was no effect on heart rate at all for Index I or II behaviors (“sequential” or “simultaneous contradiction”). As such, the most widely cited evidence used to support claims that the different behaviors in the Main and Solomon indices are equal and equivalent expressions of a unitary process of breakdown in fact suggests the opposite. Already in *Patterns of Attachment* (Ainsworth et al., 1978, p. 276), Ainsworth and colleagues were calling for the use of physiological measures to assess whether what was then called “tension movements” represented all the same degree of stress and have quite the same antecedents, butAinsworth’s question has remained largely unrecognized by the field.

The only published study we know of to have partially addressed Ainsworth’s question, not coincidently, was co-authored by Elizabeth Carlson and Alan Sroufe, foremost experts on attachment and trainers in the Strange Situation (Padrón et al., 2014). Padrón et al. (2014) express deep concern regarding the assumption that disorganized/disoriented attachment represents undifferentiated chaos, calling this a misapprehension which “has moved researchers away from attempting to examine patterns in the attachment behavior of disorganized infants” (p. 202). The researchers divided infants placed in the D classification into two groups according to whether they did or did not show fear (Index VI) or disorientation (Index VII) in the Strange Situation. They then compared the two groups with respect to affect regulation and orientation as newborns. They found that the group who displayed Index I through V behaviors had indeed been lower in affect regulation than infants who displayed Index VI and VII behaviors, suggesting that the former may be predisposed by neurological difficulties. Unfortunately, the authors did not present data regarding the converse reciprocal hypothesis, that is, whether fear and disorientation were specifically related to more insensitive caregiving. Padrón et al. (2014) also did not have data regarding whether the caregivers displayed frightening or frightened behavior to their infants. Nonetheless, in light of the Padrón et al. (2014) findings, it is perhaps not merely now of historical interest that in a passage cut to reduce the length of Main and Solomon (1990), an already over-long chapter, it was specified that Indices VI and VII were less often seen in the tapes from normative samples and were frequent characteristic of tapes from maltreated groups.

Besides conceptual problems, an impediment to inquiry into subgroups has been the issue of cell size. Too few infants are placed in the disorganized category in most samples, it is sometimes argued, to make subtype analysis possible. As a result, the field has generally regarded differentiation of the disorganized classification into additional discrete categories as a strategic mistake for the field, especially in the context of current heightened anxiety about replicability. However, it can be circumvented by significant degrees. In the last decade, the 1–9 scale used to score D has increasingly also been treated as “a continuous measure of extent of disorganization” in order “to maximize the power of the analyses” (Bureau et al., 2009, p. 270; cf. Waters & Beauchaine, 2003). Attention to disorganization as continuous rather than categorical is in line with Main and Cassidy’s (1988) forgotten proposal that the phenomenon of disorganization itself should in the first instance be regarded as a dimension, not a category. In high-risk samples, most infants will have a score on the Main and Solomon (1990) 1–9 scale of ≥3, and in many normative samples nearly half the sample may have a ≥3 score for disorganization. This prevalence of D-behav is surprisingly little discussed—a fact which has buttressed confused attempts to use assessment for D-behav as a screening tool for child maltreatment (see Granqvist et al., 2016, for a discussion). It is a function of the reification of the D/not-D boundary and the associated lack of attention to the indices themselves. Since most infants in samples with at least one risk factor display *some* behavior in the Main and Solomon indices, we suspect that disorganisation as a unitary category may be in the same position as the unresolved classification on the Adult Attachment Interview. Bakermans-Kranenburg and van IJzendoorn (2009) observe that “the unresolved classification may be less than optimally discriminating between clinical phenotypes” (p. 250), and propose that reporting results from a small number of dimensions addressing lack of resolution would represent an advance for the field if these could be successfully validated and reported alongside the overall category. As Bakermans-Kranenburg and van IJzendoorn note, such inquiry would not undermine the the incremental validity of the existing category, or the research programme that has been built up using it. Instead, it would attempt to draw distinctions and recognise differences at a different level of analysis.

For researchers who may wish to pursue this line of inquiry, we would flag one further problem with Main and Solomon’s chapters which should be taken into account: the problematic “in-but-out” status of infant caregiving behaviors towards their parent in the operationalization of disorganization. This phenomenon has been observed in the reunion behaviors of older infants and toddlers (Crittenden, 1988) and pre-schoolers and older children (Cassidy & Marvin, 1992; Main & Cassidy, 1988). However, it does not feature in the Main and Solomon (1990) indices. Yet in the mid-1990s, Main made a number of amendments to the Main and Solomon indices in an unpublished text which is distributed to those learning to code disorganization from Elizabeth Carlson. In Index VI (“direct indices of apprehension regarding parent”), Main added an “overbright greeting” as a D-behav. The logic was that a child showing caregiving to their parent when they are anxious in the Strange Situation may be regarded in a sense as contradictory to the attachment behavioral system, in which the adult is expected to serve as caregiver (D-sys). Presently, as a result, overbright and caregiving behaviors by an infant can inform a coder making a D classification. But this has occurred without differentiation from other forms of disorganization advised by Main and Solomon (1990), and without published discussion. The potential link between infant overbright behaviors and a controlling–caregiving classification later in childhood would be an interesting, testable question. But one important consequence of the invisibility of D-behav in the field’s discussions to date is that no investigation has been made of their potential developmental trajectories.

## Conclusion

Main and Solomon (1990, p. 156) warned in the last pages of the chapter announcing the protocols for coding disorganization/disorientation that treating the items in a group as a reified category can offer undue support to beliefs that there are no meaningful differences within this group. We have not found any subsequent published work that has cited this warning. More recently Sroufe and Carlson, who have trained the large majority of current coders of the disorganized/disoriented attachment classification, have argued that the concept of “disorganization” itself has misdirected researchers and clinicians (Padrón et al., 2014). In agreement with these concerns, we have attempted here to clarify the concept of disorganized attachment. In particular, we have expressed concern that the elided difference between (1) the behaviors listed in the Main and Solomon indices, (2) disruption of the attachment system, and (3) D as a taxonomic entity—where all are called “disorganization”—has caused all kinds of confusion. Main and Solomon set out to serve as cartographers of relatively new terrain, and their account has supported a convergent, international research program. However, greater conceptual clarity and the resulting awareness of complexity will, we hope, help address unrecognized issues of construct validity and their effects, and help caution against misapplications of the classification.In response to this article, we suspect that people will want to know what, terminology aside, disorganization *is*. The answer is that a set of behaviours were initially identified as representing disruptions of the Ainsworth patterns in the Strange Situation. These were grouped together by Main and Solomon as a single category. Soon after, a theory was proposed by Main and Hesse that an important cause of such behaviour was an infant’s experience of a caregiver as themselves a source of alarm. It seems likely that this hypothesis accounts for a good deal of what observers see in the Strange Situation among infants classified as disorganized. But it remains untested whether all the behaviours listed by Main and Solomon represent fear to the same extent and in the same way. We hope that distinctions between levels of analysis will help facilitate such work. Without having tested this question, we cannot feel secure in assuming that disorganization carries the same level of risk when shown by maltreated children, those who have experienced repeated separations from the caregivers, children of parents suffering from an affective disorder, and those in normative community samples (Solomon & George, 2016).

With greater conceptual clarity, we hope, the different aspects of the phenomena discussed under the rubric of “disorganization” will more readily remain in sight and sustain attention from researchers and clinicians, and they will more readily be found when they are looked for in the course of both research and clinical discussions and debates.
